# Interaction Domain of Glycoproteins gB and gH of Marek's Disease Virus and Identification of an Antiviral Peptide with Dual Functions

**DOI:** 10.1371/journal.pone.0054761

**Published:** 2013-02-06

**Authors:** Xiao-Jing Chi, Yi-Xin Lu, Peng Zhao, Chuan-Gen Li, Xiao-Jia Wang, Ming Wang

**Affiliations:** 1 Key Laboratory of Zoonosis of Ministry of Agricultrure, College of Veterinary Medicine, China Agricultural University, Beijing, People's Republic of China; 2 Department of Preventive Veterinary Medicine, College of Veterinary Medicine, Shandong Agricultural University, Shandong, People's Republic of China; Centre National de la Recherche Scientifique, Aix-Marseille Université, France

## Abstract

Our previous study reported that both glycoproteins gB and gH of the herpesvirus Marek's disease virus (MDV) contain eleven potential heptad repeat domains. These domains overlap with α-helix-enriched hydrophobic regions, including the gH-derived HR1 (gHH1) and HR3 (gHH3) and gB-derived HR1 (gBH1) regions, which demonstrate effective antiviral activity, with 50% inhibitory concentrations (IC_50_) of less than 12 µM. Plaque formation and chicken embryo infection assays confirmed these results. In this study, biochemical and biophysical analyses detected potential interactions between these peptides. gHH1, gHH3, and gBH1 were found to interact with each other in pairs. The complex formed by gHH3 and gBH1 showed the most stable interaction at a molar ratio of 1:3, the binding between gHH1 and gBH1 was relatively weak, and no interaction was observed between the three HR peptides. These results indicate that gHH3 and gBH1 are likely the key contributors to the interaction between gB and gH. Furthermore, each HR peptide from herpesvirus glycoproteins did not effectively inhibit virus infection compared with peptides from a class I enveloped virus. In this report, the HR mimic peptide modified with a double glutamic acid (EE) or a double lysine (KK) at the non-interactive sites (i.e., solvent-accessible sites) did not noticeably affect the antiviral activity compared with the wild-type HR peptide, whereas tandem peptides from gH-derived gHH1 and gB-derived gBH1 (i.e., gBH1-Linker-gHH1) produced efficient antiviral effects, unlike the individual peptides. The proposed interpretation of inhibition of entry has been addressed. Our results support the hypothesis that the interaction domain between glycoproteins gH and gB is a critical target in the design of inhibitors of herpesvirus infection.

## Introduction

Marek's disease (MD) is a communicable viral lymphoproliferative disease of chickens. It is caused by the oncogenic Marek's disease virus (MDV). MDV is classified as an alphaherpesvirus according to DNA sequence homology and genome organization, although its biological properties are more similar those of gammaherpesviruses [1], [2]. Due to its unique properties, MDV has long been of interest as a model organism [3]. Recent advances in MDV genetics and the sequencing of the chicken genome aided by functional genomics have improved our understanding of lytic MDV replication and the mechanisms leading to latency and tumor formation [4], [5]. Most of the existing studies on MDV have focused on non-oncogenic MDV strains as a vaccine for preventing tumors [6], [7], [8]. The underlying mechanisms responsible for MDV entry into cells remain not well understood.

Enveloped viruses infect host cells by fusion of viral and target membranes. Membrane fusion between a herpesvirus and a host cell is mediated by one or more viral fusion glycoproteins and their conformational change. The fusion glycoproteins belong to either class I, class II or the newly described class III, which depend upon their arrangement on the virion surface and the structure and location of a short stretch of hydrophobic amino acids called the fusion peptide within the protein, which induces the initial lipid destabilization that culminates in fusion [9], [10]. Three glycoproteins that are essential for entry, gB and the gH-gL heterodimer, are conserved throughout the alphaherpesvirus family [11], [12]. For herpes simplex virus type-1 (HSV-1) and HSV-2, syncytium formation requires the expression of gH–gL, gD and gB [13], [14], [15], and gB and gH–gL play important roles in the primary fusion events that occur during egress of the capsid from the nucleus of infected cells [16]. In contrast, varicella-zoster virus (VZV) gH-gL causes cell-to-cell fusion [17]. Both MDV and VZV do not have a glycoprotein gD homologue. It was recently shown that HSV-1 gB and gH–gL interact with each other concomitant with fusion and that this interaction is triggered by binding of gD to its cellular receptor1 [18], [19]. At the same time, the crystal structure of gB indicates that gB is likely a fusogen protein for HSV fusion [20]. gB is assumed to be directly involved in bringing the viral and cellular membranes together through a triggered conformational change. As members of the newly formed class III group of fusion glycoproteins, herpesvirus gB proteins share similar individual domain structures and harbor the central three-stranded coiled-coil of the class I proteins. During the fusion process, gB may function cooperatively with gH–gL and cannot function alone [20], [21]. The crystal structure of the gH ectodomain bound to gL is an unusually stable complex with unique characteristics [22]. gH shares certain features with class I fusion proteins [15], [23], [24].

Both gH and gB have several hydrophobic fusogenic domains to block virus entry or disrupt cellular membranes in a dose-dependent manner, such as heptad repeat (HR) regions [25], [26], [27], [28]. We have previously determined the biological functions of specific regions of MDV gH and gB and found that gHH1, gHH3, and gBH1 overlap with some α-helix-enriched regions. These peptides exhibit antiviral activities during different stages of membrane fusion [29]. Here, we used GST pull-down, gel filtration and circular dichroism (CD) spectroscopy analyses to study the interactions of these peptides. A potent antiviral inhibitor peptide with dual roles was then designed and evaluated.

## Results

### Design of HR-derived peptides and prediction of interactions

**Figure 1:**
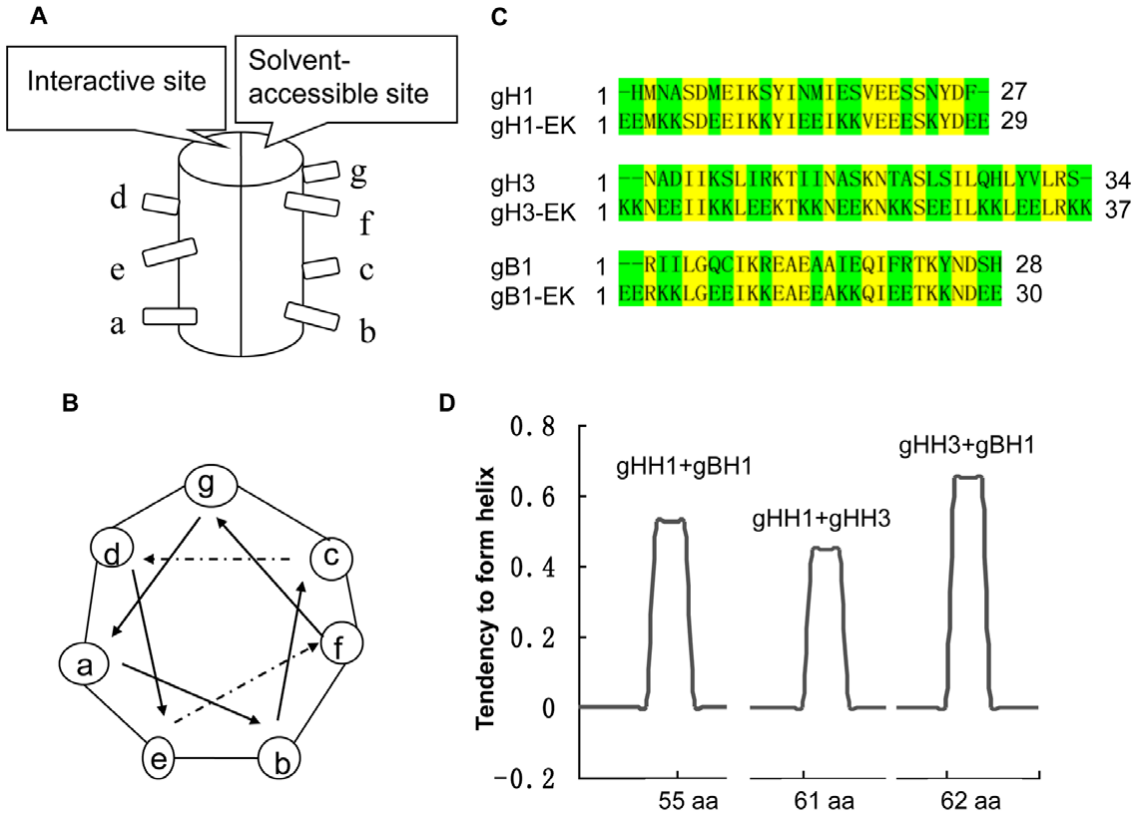
HR α-helix wheel and interaction prediction. (a) Standard heptad repeat motif has interaction and solvent-accessible sites. (b) In the heptad repeat of α-helix wheel, positions *a, d* and *e* are the interactive sites and positions *b, c, f,* and *g* are the solvent-accessible sites. (c) Amino acid sequences of the three peptides and corresponding EK modified peptides with underlines are *a, d,* and *e* sites, respectively. (d) Predict-protein computational (https://www.predictprotein.org) was used to predict the tendency to form complex for mixed peptides in pairs. The tendency of gHH1 and gBH1 to form complex is 52.5%, of gHH1 and gHH3 is 44.8%, and of gBH1 and gHH3 is 64.7%.

The primary structure of each helix consists of a heptad repeat composed of seven residues denoted by (a-b-c-d-e-f-g)n. The nonpolar residues occupy positions *a, d* and *e*, forming a hydrophobic surface. Positions *b, c, f* and *g* are solvent-exposed and often populated by polar residues (Figure 1A and 1B). We addressed the design of inhibitors in two steps: (i) analysis of the gHH1, gHH3, and gBH1 motifs; and (ii) design of mutations aimed at improving the helical interaction. In this study, we developed HR-based EK modification peptides of gHH1-EK, gHH3-EK, and gBH1-EK. For EK modified residues at the solvent-accessible sites of *b, c, f* and *g* (Figure 1C), a series of systematic replacements with double hydrophilic glutamic acid (E) or lysine (K) were introduced to enhance the α-helicity of the peptides by intrahelical salt bridges [30]. At the same time, the interaction sites (*a, d, e*) of these peptides remained intact. In addition, a series of computational tools (ExPASy-Coils, PIE, PredictProtein) were used to predict the tendency to form a complex of peptides. The results showed that the binding between gHH3 and gBH1 is stronger than other pairs of peptides (Figure 1D).

### Interaction of glycoproteins gB and gH in infected cells

**Figure 2:**
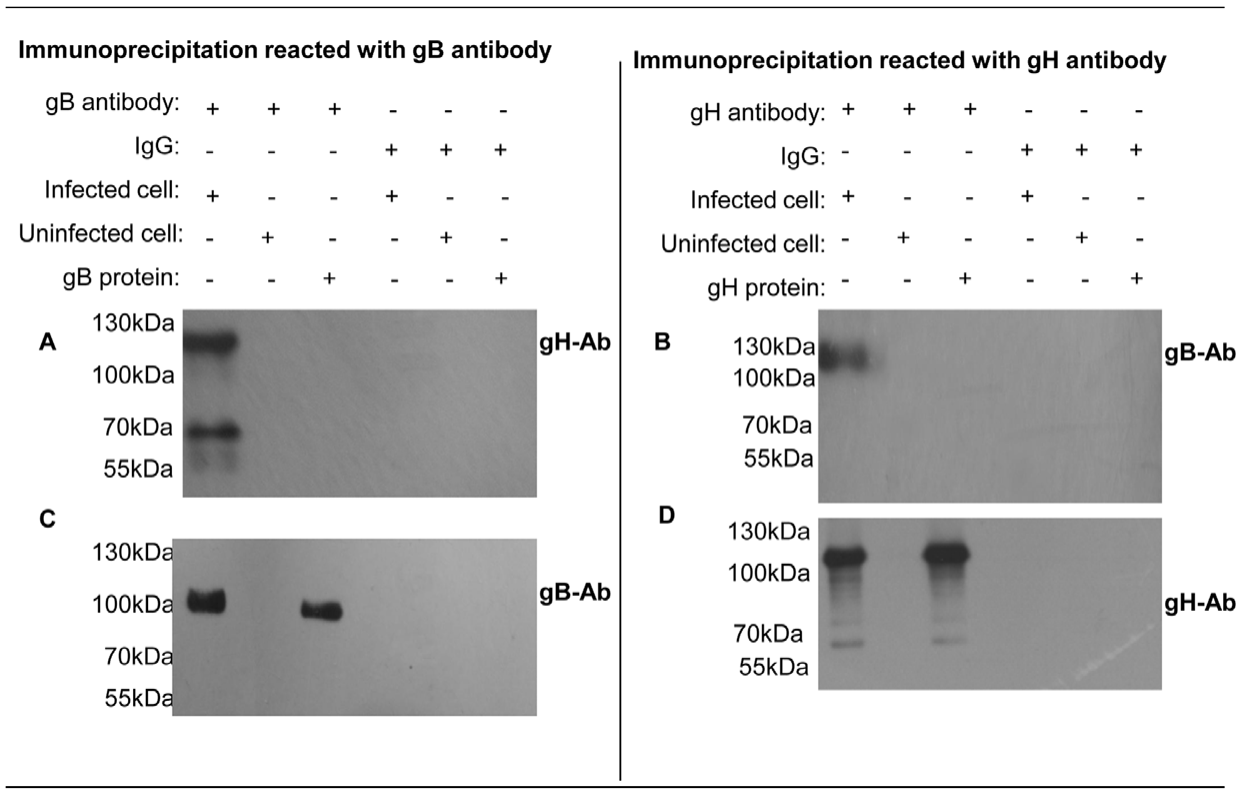
Immunoprecipitation results. Right: MDV infected or mock infected cell lysates were collected with gB antibody. Immunoblots reacted with gH antibody (a) and gB antibody (c). From left to right, MDV infected cell lysates were mixed with gB antibody, uninfected cell lysates were mixed with gB antibody, gB protein was mixed with gB antibody, infected cell lysates mixed with IgG antibody, uninfected cell lysates were mixed with IgG antibody, gB protein was mixed with IgG antibody; Left: MDV infected or uninfected cell lysates were collected with gH antibody. Immunoblots reacted with gB antibody (b) and gH antibody (d). From left to right, MDV infected cell lysates were mixed with gH antibody, uninfected cell lysates were mixed with gH antibody, gH protein was mixed with gH antibody, infected cell lysates mixed with IgG antibody, uninfected cell lysates were mixed with IgG antibody, gH protein was mixed with IgG antibody.

Membrane fusion of a herpesvirus envelope with a cellular membrane employs conserved core fusion machinery. The machinery involves glycoprotein gB and the non-covalently associated heterodimer gH–gL [11], [12], [31]. gB is highly conserved among all members of the family *Herpesviridae* and is involved in virus attachment, penetration and cell-to-cell spread [31]. In this study, the immunoprecipitation revealed the interaction between gB and gH in MDV -infected cells. Primary chicken embryo fibroblast (CEF) cells were infected with MDV for 5 days, and the infected cell monolayers incubated with either anti-gB or anti-gH antibodies, followed by western blot analysis with either anti-gH or anti-gB antibodies individually. As shown in Figure 2, the gH antibody can pull down gB and vice versa. In a positive control, immunoprecipitation reacted with anti-gB or anti-gH antibodies. Western blotting analysis with the same antibody gave identical bands, indicating that the interaction between gB and gH is involved in the virus infection.

### Interaction between pairs of peptides

**Figure 3:**
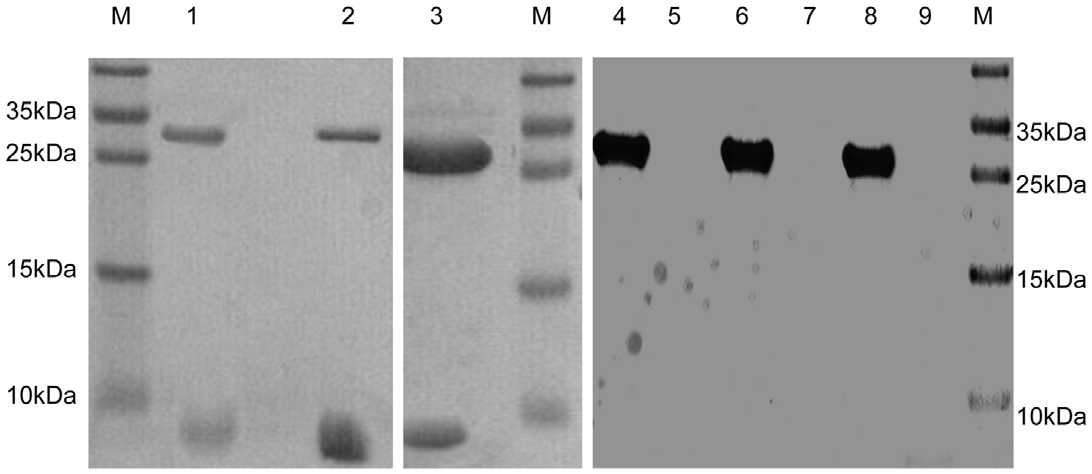
GST pull-down results. Peptides of gHH1 or gHH3 were incubated with GST-fused gBH1 protein and GST-fused gHH1 protein respectively, protein mixtures were collected with Sepharose 4B beads, and SDS-PAGE was then analyzed. From left to right, 1, GST-gBH1 as bait mixed with gHH3; 2, GST-gBH1 as protein mixed with gHH1; 3, GST-gHH1 as bait mixed with gHH3; 4, GST scramble (gBH1 random peptide) as bait mixed with gHH1; 5, free peptides gHH1 without bait mixed with glutathione-Sepharose beads; 6, GST scramble (gBH1 random peptide) as bait mixed with gHH3; 7, free peptides gHH3 without bait mixed with Sepharose beads; 8, GST scramble (gHH1 random peptide) as bait mixed with gBH1; 9, free peptides gBH1 without bait mixed with Sepharose beads.

It is widely accepted that two peptides corresponding to the HR domains of a fusion glycoprotein from a class I enveloped virus can interact to form a six-helix bundle structure [32]. Herpesviruses encode at least two glycoproteins, gB and gH, which contain several HR domains, and neither shows efficient antiviral activities [25], [29], [33]. In this paper, biochemical and biophysical analyses were used to detect potential binding between the domains. We determined that MDV gH-derived gHH1 and gHH3 and gB-derived gBH1 can interact in pairs. The GST pull-down assays shown in Figure 3 indicate that GST-gBH1 can pull down either gHH3 or gHH1, and GST-gHH1 can also pull down gHH3. These observations also demonstrate that the three peptides can be considered effective drug target candidates for preventing the interaction between gB and gH.

### Competitive interaction between HR peptides and conformational change

**Figure 4:**
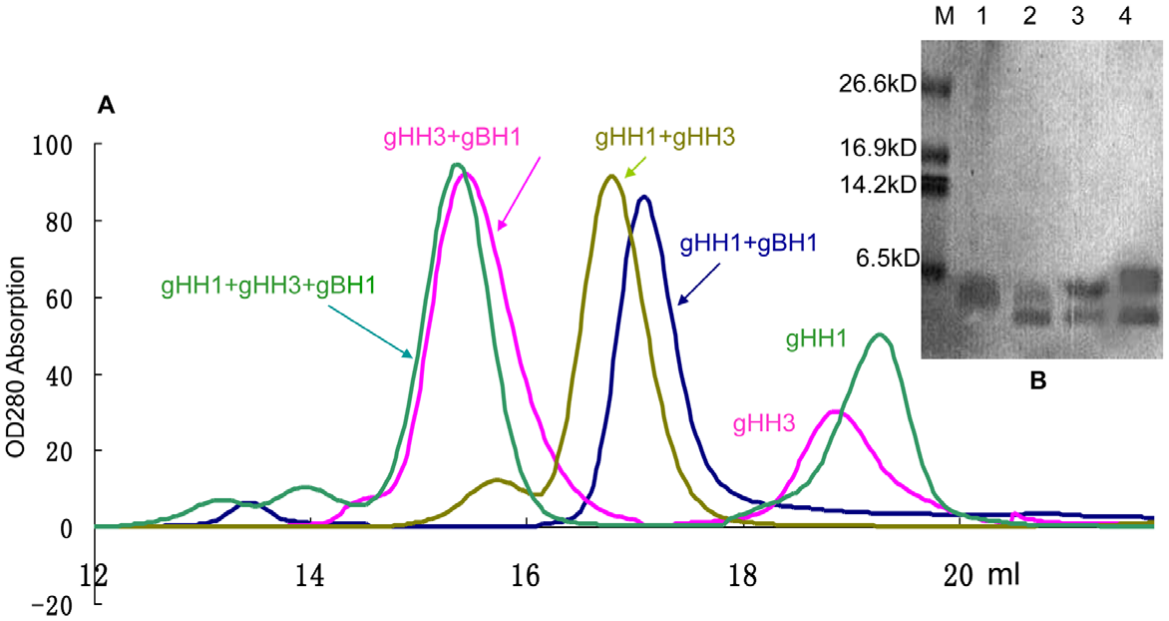
Gel filtration analysis. (a) The peptides were loaded onto the Superdex G75 column in a buffer solution of 20 mM Tris-HCl, pH 8.0. The molecular mass of peak protein was estimated by comparison with the protein standards running on the same column. The clear peak of complex of gHH1 with gHH3 at the equimolar concentration occurred at about 7.0 kDa. This molecular mass matches the approximate sum of one molecule of gHH1 and one molecule of gHH1, indicating the formation of the heterodimer structure. The peak of gHH1 with gBH1 at the equimolar concentration occurred at about 6.5 kDa, indicating the formation of the heterodimer structure. The peak of gBH1 with gHH3 at the equimolar concentration occurred at about 14.5 kDa, indicating the heterotetramer state, matches the approximate sum of three gBH1 and one gHH3, and separate peak with low molecular weight is a gHH3 analyzed in Figure 4 (b) lane 1. The main peak of gBH1, gHH3 and gHH1 at a molar ratio of 3∶1∶1 appeared same as a complex of gBH1 with gHH3. (b) SDS-PAGE analysis. All the peaks of the samples were collected for 5 min at 100°C and then analyzed by 18% SDS-PAGE. From left to right, Marker; lane 1, separate peak with low molecular mass while gHH3 and gBH1 peptides were mixed (i.e. gHH3); lane 2, main peak formed the mixture of gBH1 with gHH3; lane 3, main peak formed the mixture of gHH1 with gBH1; lane 4, main peak formed the mixture of gHH1 with gHH3.

Our previous study based on mass spectrometry (MS) and gel filtration showed that the molecular weights of gHH1, gHH3 and gBH1 are 3184, 3795 and 3303 Da, respectively. In addition, the gBH1 peptide forms a homotrimeric structure with a molecular weight of 10.7 kDa. gHH3 adopts a monomer formation with a molecular weight of 3.8 kDa, and gHH1 forms a homotetramer with a molecular weight of approximately 12.1 kDa [29]. Gel filtration was used to confirm the interactions between peptides. The profiles of the peptide mixtures showed a significant change with the appearance of a new peak. As shown in Figure 4 and Table 1, the mixtures of gHH1 with gHH3 and gHH1 with gBH1 formed a heterodimer. The mixture of gHH3 with gBH1 formed a tetramer, which consisted of three molecules of gBH1 and one molecule of gHH3. GST pull-down and gel filtration assays support the proposal that each pair of three peptides is capable of interacting with each other. When the three peptides were mixed under the same experimental conditions, the peak on the Superdex column showed the same value as the mixture of gHH3 with gBH1. Therefore, the combination of gHH3 and gBH1 is hightly stable and can prevent the formation other forms of other heteromers, such as gHH1 with either gHH3 or gBH1.

**Table 1:** Calculated complex construction of peptides in aqueous solution.

Peptides	Molecular mass	Molecular state
gHH1	12.1 kDa	Tetramer
gHH3	3.8 kDa	Monomer
gBH1	10.7 kDa	Trimer
gHH1+gHB1	6.5 kDa	**1** gHH1+**1** gHB1
gHH1+gHH3	14.5 kDa	**3** gHH1+**1** gHH3
gHH3+gHB1	7.0 kDa	**1** gHH3+**1** gHB1

The mixtures of gHH1 with gHH3, and gHH1 with gBH1 formed a heterodimer; the mixture of gHH3 with gBH1 formed a tetramer, which consists of three molecules of gBH1 and one molecule of gHH3.

**Figure 5:**
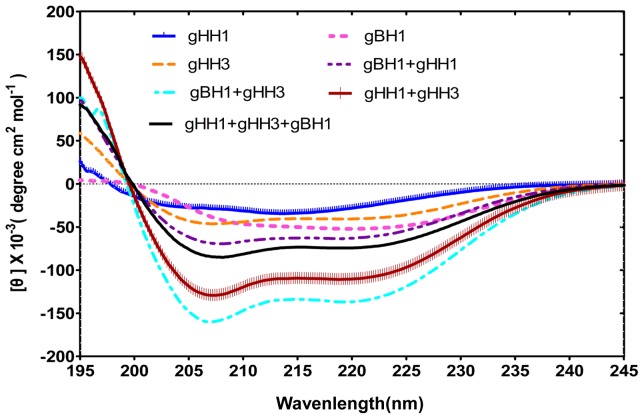
CD results of individual or mixed peptides at the equimolar concentration. Structural analysis of three individual peptides and mixed peptides at the equimolar concentration. Peptides were measured at concentration of 10 μM in PBS with 25% 2,2,2 trifluoroethanol (TFE). CD analysis showed that all the peptides adopt standard α-helical structure, and the mixture of gHH3 with gBH1 showed most obvious tendency to form helix.

**Figure 6:**
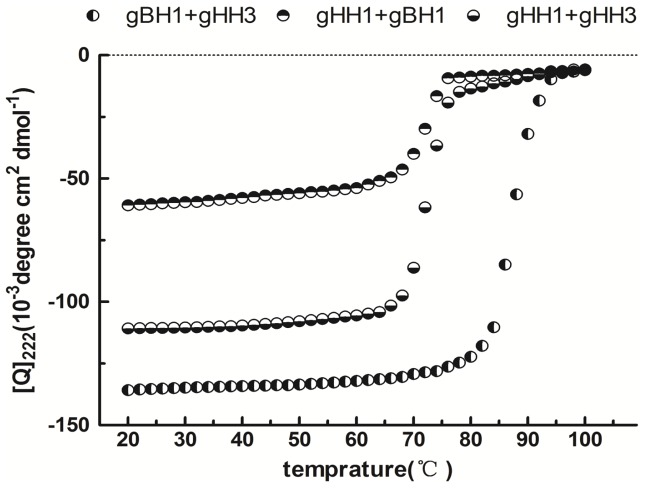
Thermal denaturation curves of mixed peptides. The curves showed the thermal stability of the peptide mixture was assessed using CD by monitoring the ellipticity at 222 nm as a function of temperature at 30 μM concentration in PBS with 25% TFE. The mixtures of gBH1 with gHH3, gHH1 with gBH1, gHH3 with gHH1 formed stable helical structures, with the apparent melting temperature (Tm) of 85°C, 70°C, and 65°C.

Circular dichroism (CD) was performed to characterize the conformational change of the peptide mixtures. The solvent 2,2,2 trifluoroethanol (TFE) is widely used in conformational studies because it promotes intramolecular hydrogen bonds despite intermolecular interactions with water molecules. Moreover, because TFE reduces the polarity of the solution, the environmental changes produced by the peptides resemble those of the native sequences during the membrane fusion process. CD analysis revealed that all of the peptides adopted a standard α-helical conformation with double minima at 208 nm and 222 nm in a PBS solution in the presence of 25% TFE (Figure 5). If there is no interaction between peptides, each peptide should maintain its original structure, and the spectrum values would consist of the average of the constituent peptides. In contrast, if peptides do interact, a structural change will take place and produce a significant change in the spectrum. Figure 5 shows the CD results for individual and mixed peptides. The significant differences between individual and mixed peptides indicate that the peptides do interact to produce a conformational change. The CD results correlate well with those of gel filtration experiments. An obvious change occurred with the mixture of gHH3 with gBH1, indicating that this combination has the highest probability of forming coiled-coils. Furthermore, the CD spectrum value in the buffer solution without TFE in Figure 6 clearly demonstrates that the thermal denaturation temperature (Tm) of the gBH1 and gHH3 complex (greater than 85 °C) was higher than that of the mixtures of gHH1 with gHH3 (approximately 65 °C) or with gBH1 (approximately 70°C), indicating that the gBH1-gHH3 complex is more stable than others. In comparison, the individual peptides formed typical helical structures with Tms of approximately 45–60°C [25], [26], [27], [28].

### Effects of HR-based peptides on virus infection

**Figure 7:**
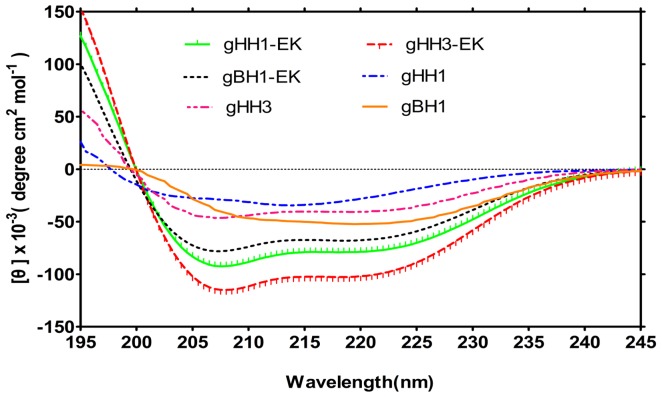
CD spectra of modified peptides analogues. Experiments were performed by comparing the spectrum of the peptides pre-modified and post-modified at the desired concentrations. Spectra were measured at concentration of 30 μM in PBS with 25% TFE. The modification peptide showed a higher probability of forming helix structure than the original mother peptide.

**Figure 8:**
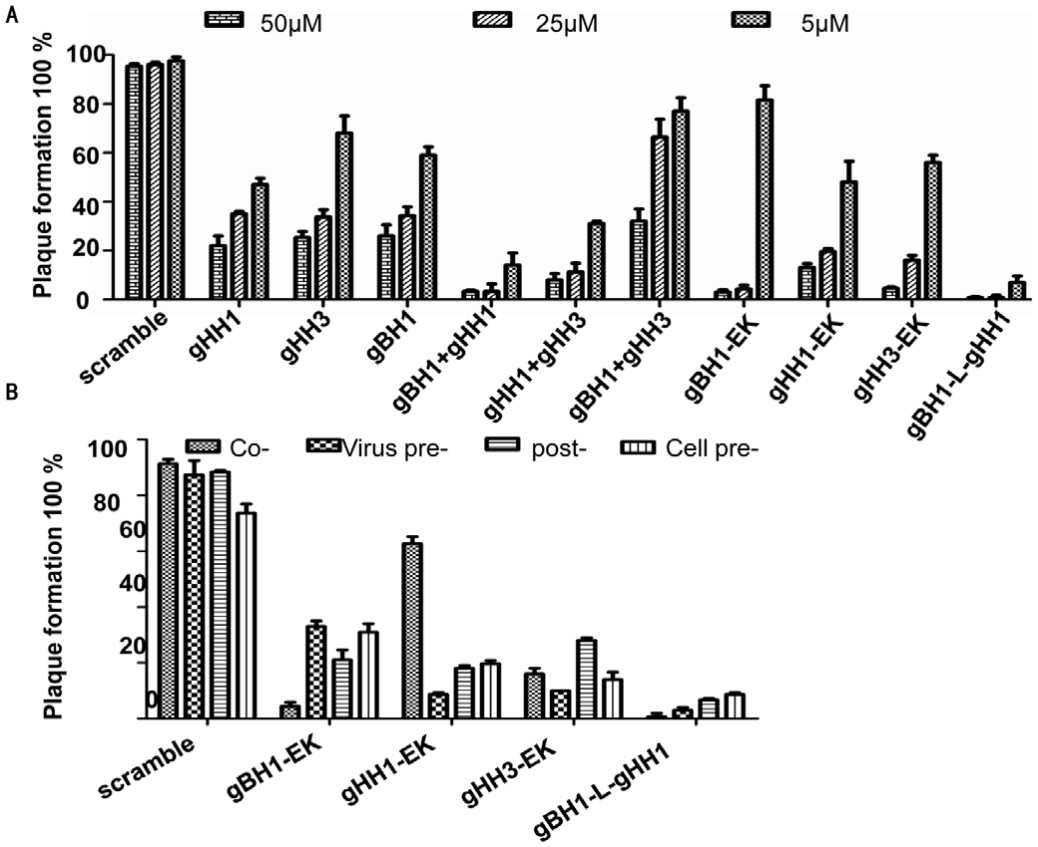
Peptide inhibition of MDV infectivity. (a) CEF cells were incubated with individual peptide or mixed peptide in pair at concentration of 50 μM, 25 μM, 5 μM in the presence of the viral inoculums for 5 days at 37°C, the percentage of plaque formation was analyzed. (b) Cells were exposed to peptides at a concentration of 25 μM either prior to infection (Cell pre-exposure), during attachment and entry (Co-exposure), after virus penetration (Post-exposure) or, alternatively, the virus was pre-incubated with peptides before addition to the cells (Virus pre-incubation). The cells were then incubated for 5 days at 37°C in 2% DMEM. Experiments were performed in triplicate and the percentage of plaque formation was calculated with respect to scramble-peptide control experiments. Error bars represent standard deviations.

To confirm that none of the peptides were toxic to CEF cells, monolayers were exposed to different concentrations (25, 50, and 100 μM) of each peptide for 24 hrs, and cell viability was characterized by an LDH assay. No significant difference in viability was observed between untreated cells and cells exposed to the peptides (data not shown). To assess the effects of the three peptides on MDV infectivity, we inoculated CEF cells with MDV at 37°C for 5 days [29], [34], [35] in the presence or absence of each peptide under a range of different conditions as described in Materials and Methods. The activity of gHH1-EK, gHH3-EK and gBH1-EK against MDV was assessed by plaque reduction assay. The peptides modified with EK produced two-fold lower IC_50_ values than the original peptides, although EK modification enhanced α-helix formation (Figures 7 and 8B). Therefore, the peptides from a single glycoprotein are not sufficient to inhibit virus infection during different entry stages even when mutated to enhance coiled-coil formation.

### Discovery and evaluation of antiviral peptides with dual functions

It has been reported that HR peptides from herpesvirus glycoproteins cannot effectively inhibit virus infection compared with those of class I enveloped viruses [25], [29], [33]. The gHH1, gHH3 and gBH1 peptides showed effective antiviral activity, with approximately 60%–70% inhibition at a concentration of 25 μM [29], whereas the HR peptide derived from a class I enveloped virus completely inhibited virus entry at the same concentration [32], [36], [37]. In this study, pairs of peptides were mixed to examine their influence on plaque formation induced by MDV infection, and the peptides mixtures showed a dose-dependent inhibition of plaque formation (Figure 8A). The mixture of gHH1 with either gBH1 or gHH3 showed greater antiviral activity than the individual peptides. The situation was even more apparent for the mixture peptides of gBH1 with gHH1 and the tandem peptide gBH1-Linker-gHH1, which produced an approximately 10–30-fold increase in antiviral activity compared with the individual peptides. Furthermore, gBH1-Linker-gHH1 inhibited plaque formation by approximately 90% at a concentration of 5 μM and during four different stages of MDV infection (Figure 8A and 8B). These results support our proposition that the tandem peptide composed of two HR domains of gB and gH can be employed as an improved strategy to develop a potent antiviral inhibitor of herpesvirus entry.

Under the same experimental conditions, the mixture peptides gBH1 and gHH3 had little effect on plaque formation, whereas the mixture of three peptides (IC_50_  = 4.7 μM) showed the same level of antiviral activity as gHH1 (IC_50_ = 4.1 μM) (Figure 8A). These findings further supports conclusion that gBH1 and gHH3 do interact to form a stable complex.

## Discussion

During enveloped virus infections, membrane fusion is of fundamental importance and interest, and the process is controlled by one or more viral surface proteins that undergo conformational changes to drive membrane fusion [9]. The initial apposition step is followed by fusion of the outer leaflets of membranes (the hemifusion step), leading to the formation of a transient fusion intermediate, which evolves into the fusion of inner leaflets and the formation of a pore and mixing of the internal compartments of both fusion partners [38], [39]. The fusion peptide is involved in all fusion events and plays a key role in the conformational changes leading to the interaction of the fusion peptide with the target membranes and following the initial peptide-membrane interactions. Herpesvirus gB and gH–gL have been considered core members of the glycoprotein complex during membrane fusion [11], [12], [31]. Several studies have suggested that gH–gL acts as a fusogen glycoprotein, possibly mediating the hemifusion step [40]. Candidate fusion peptides corresponding to gH hydrophobic regions has been shown to bind lipids [41], [42], [43], [44]. Furthermore, gH–gL can promote fusion of the virion envelope with the outer nuclear membrane during viral egress [16]. Although these studies suggested that gH–gL has fusogenic capacity, the recently solved crystal structures of the gH-gL ectodomains demonstrated the gH–gL complex has no structural homology to any known fusion proteins [22]. The structural data support a model in which gH–gL acts not as a fusogen but primarily as a regulator of fusion through interactions with gB. However, the interaction domain between gB and gH during the virus entry processis not well characterized, although the 3D structure of gH and gB and their receptor have been determined [20], [22]. In the present study, immunoprecipitation assays indicated that glycoproteins gH and gB can interact during virus infection (see Figure 2).

Both gH and gB contain several α-helix-enriched hydrophobic regions in the ectodomain that clearly play an important role in the fusion process [29]. Biochemical and biophysical analyses were used to detect potential interactions between these peptides and demonstrate that the HR mimics gHH1, gHH3, and gBH1 can interact with each other in pairs with an increasing tendency to form α-helix structures in compared with the individual peptides (Figures 3, 4, 5). Amino acid alignments were employed to compare the corresponding domains of gHH1 with those of other alphaherpesviruses. No significant antiviral activity was found in published reports. Whereas gHH3 is homologous to HR1 of HSV-1 gH, which shows potent antiviral activity in infectivity assays [25], gBH1 is homologous to HSV-1 gB406–433 [45], which was unable to induce lipid mixing and did not significantly inhibit infection. MDV glycoprotein-derived peptides showed different antiviral functions from the corresponding domains derived from HSV-1 gH. Additional issues concerning the similarities and differences between the membrane fusion mechanisms of MDV and other α-herpesviruses should also be addressed. In this paper, gHH1 and gHH3 of MDV gH showed a possibility of forming a heterodimer. The Tm of the complex, a measure of its thermodynamic stability, was approximately 65°C, whereas its stability would be altered by gB to form a stability-enhanced heterotetramer of gHH3 and gBH1, with a Tm of greater than 85°C, and/or a heterodimer of gHH1 and gBH1 mixture with Tm of 70°C (Figure 6). Computational tools predicted that gHH3 and gBH1 would interact strongly (Figure 1). This prediction was confirmed by gel filtration and CD. The complex formed by gHH3 and gBH1 showed the most stable binding at a molar ratio of 1∶3, whereas an interaction between the three HR peptides did not occur under the current experimental conditions. The complex peak containing gHH3 and gBH1 appeared on a Superdex column when the three peptides were mixed (Figure 4). Furthermore, the mixture peptides of gBH1 and gHH3 had little effect on plaque formation, whereas the mixture of the three peptides showed the same level of antiviral activity as gHH1 in virus infectivity assays (Figure 8A, Table 2). These results are consistent with those of *in vitro* protein interaction assays and further support the conclusion that gBH1 and gHH3 do interact to form a stable complex during virus infection.

**Table 2:** The antiviral activity IC_50_ of peptides.

Peptides	IC50	peptides	IC50
gHH1-EK	4.0±0.12μM	gHH1	4.1±0.26μM
gHH3-EK	6.3±1.16 μM	gHH3	12.2±0.28 μM
gHB1-EK	8.7±1.29μM	gBH1	9.0±0.27μM
gHH1+gHB1	0.4+0.06μM	gHH1-Linker-gHB1	0.3±0.03 μM
gHH1+gHH3	1.6±0.31μM	gHH1+gHH3+ gHB1	4.7±0.81μM
gHH3+gHB1	>25 μM	scramble	>500μM

Each sample was tested in triplicate, and the data were presented in means ± SD.

For type I fusion protein, fusogenic glycoproteins possesses two heptad repeat domains [32]. HR1 and HR2 can form of a six-helix bundle structure, which is known as the virus fusion central core and is believed to bring the viral and cellular membranes closer together. The synthetic peptides corresponding to the C-terminal HR are generally more active in inhibition assays compared with peptides corresponding to the N-terminal HR [32], [36], [37]. This observation is in contrast with our results with MDV and other herpesviruses [25], [29], [33], in which three N-terminal HRs show a higher probability of forming coiled-coils (Figures 1 and 5) and potent antiviral activity (Figure 8). The three peptides gHH1, gHH2, and gBH1 efficiently inhibited plaque formation, with an IC_50_ of less than 12 µM, and gHH1 blocked viral infection in both virus co-treatment (co) and post-treatment (post) assays [29]. There are several mechanisms by which peptides could inhibit MDV entry. These peptides could disrupt the structure of gB. If this were true, the peptides would be expected to be virucidal in virus pre-treatment. However, here, gHH1 and gHH3 demonstrated no or low virucidal activity. It is also possible that gHH1 and gHH3 could either prematurely trigger or inhibit a conformational change in the gB molecule that is required for entry. Finally, the peptides may act by blocking a protein-protein interaction. Moreover, HR peptides from MDV and other herpesviruses showed less inhibition than those of class I enveloped viruses [25], [33], [36], [37]. However, any individual HR-derived peptide seems to play no significant role in preventing herpesvirus entry. Therefore, we considered a so-called tandem strategy and constructed the gBH1-Linker-gHH1 peptide, which was obtained from different glycoproteins and connected by a linker (SGGRGG). The peptide gBH1-Linker-gHH1 did indeed increase its capacity to inhibit infection during specific stages of virus entry, including a post-attachment entry step, with an IC_50_ of less than 0.3 µM. It seems that the peptide gBH1-Linker-gHH1 would likely be useful as a preventive agent or as a microbicide. However, the antiviral activity of EK-modified peptides was within the same order of magnitude as the parent HR peptide (Figure 8A and 8B), although the probability of forming coiled-coils was increased (Figure 7). These results emphasize that the association and interaction domains of glycoproteins gB and gH are a critical consideration in designing herpesvirus inhibitors.

**Figure 9:**
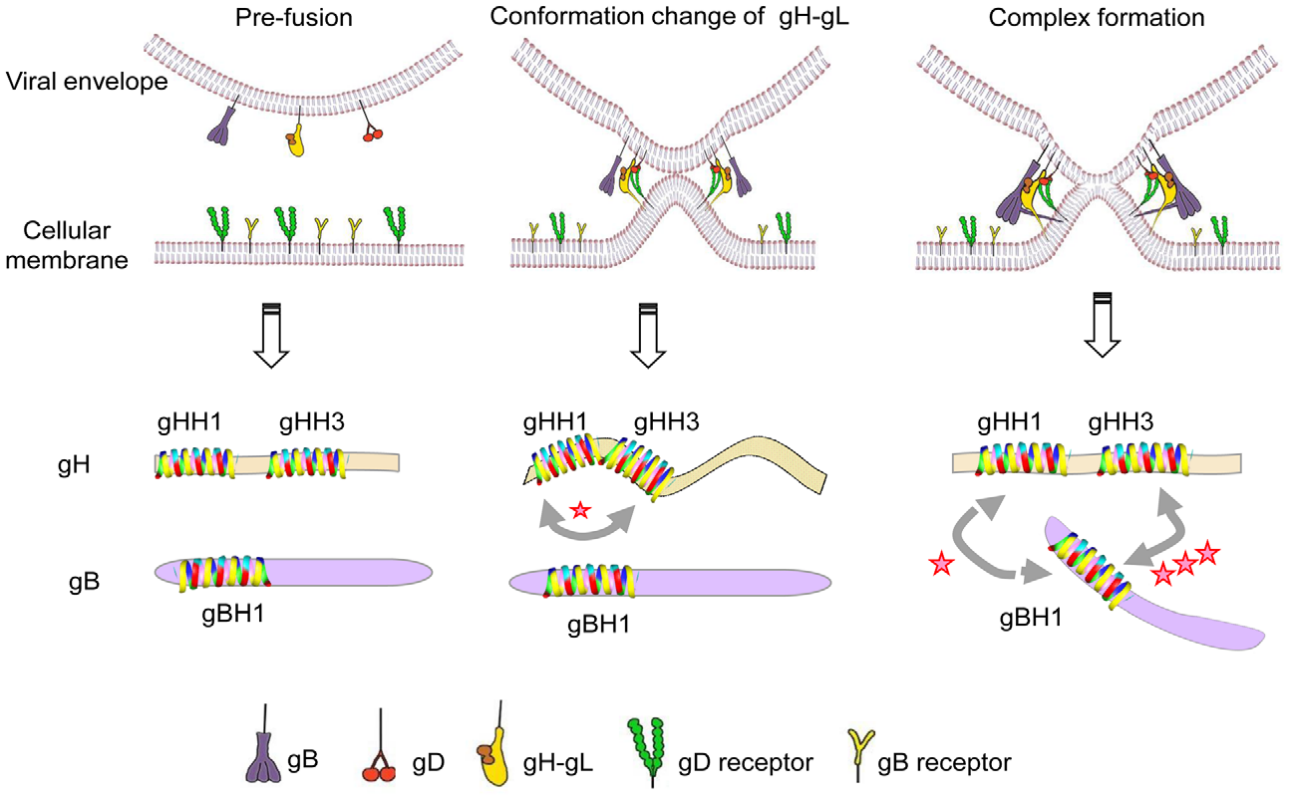
Schematic illustration of the proposed mechanism. The diagram showed the proposed mechanism in which the interaction of gHH3 and gBH1 improved virus entry. It is broadly accepted that gB was recruited to the core gH-gL complex and consequently induced HSV-1 membrane fusion (**top**); At an earlier stage of membrane fusion, the gHH1 and gHH3 of gH glycoprotein remain connected in the absence of gB. Since gB was recruited to the gH-gL complex, an linking between gHH1 and gHH3 was completely depressed and displaced by the overwhelming interaction of gBH1 with gHH3, and/or gBH1 with gHH1. The corresponding membrane fusion was triggered and improved (**bottom**).

In steps in common with HSV-1, upon receptor binding, conformational changes in MDV gH–gL activate its interaction with gB, which acts as the viral fusogen [12], [14], [15]. Figure 9 shows the proposed details based on the initial findings for HSV-1. At an early stage of membrane fusion, the gHH1 and gHH3 of the gH glycoprotein interact in the absence of gB. When gB is recruited to the gH-gL complex, the interaction between gHH1 and gHH3 is inhibited and displaced by the interaction of gBH1 with gHH3 and/or with gHH1. Subsequently, membrane fusion occurs at a later stage. It is worth noting that the association and dissociation between HR domains of gH and/or gB can occur dynamically during virus entry. In this paper, the peptides gHH1, gHH2, and gBH1 show a dose-dependent inhibition of plaque formation, and the tandem peptide of gBH1-Linker-gHH1 showed the most effective antiviral activity compared with other peptides tested (Figure 8). The gBH1-Linker-gHH1 peptide may play a role at an earlier stage of membrane fusion by blocking the interaction between gHH1 and gHH3 and at a later stage by competitively blocking the interaction between gBH1 and gHH3. From this standpoint, gHH3 would be a potential and valuable candidate for antiviral drug development.

Our study identified the interaction between glycoproteins gH- and gB-derived peptides and investigated their corresponding functions related to virus entry, based on which newly designed antiviral inhibitors with dual functions activity have been discovered and evaluated. This paper provides a scientific basis for the interpretation of virus entry mechanisms and the development of candidate drugs against MDV. Many details need to be further explored, such as how the peptides associate with glycoproteins gH or gB to block the conformational changes that are critical for membrane fusion or binding to receptors and the corresponding dynamics of these events. Nevertheless, the interaction between gHH3 and gBH1 during virus entry has been identified as a critical process.

## Materials and Methods

### Prediction and analysis of fusogenic regions

The availability of computational software (ExPASy) prompted us to analyze the different domains of gH and gB of MDV strain RB1B in detail as a putative membrane-interacting region [22]. Our previous study reported that both glycoproteins gB and gH of MDV contain eleven potential heptad repeat (HR) domains. Three peptides were chosen for this study, including gH-derived HR1 (gHH1) and HR3 (gHH3), located at amino acid residues 277 to 303 and 396 to 429, respectively, and gB-derived HR1 (gBH1), located at residues 340 to 36,. We chose the PIE software and PredictProtein to analyze the interaction potential between the peptides. In some experiments, tandem peptide pairs connected by six amino acid linker (SGGRGG) (i.e., gBH1-Linker-gHH1) were constructed. For MDV gHH1, gHH3, and gBH1, a series of systematic replacements with hydrophilic glutamic acid (E) or lysine (K) residues were made at the *b, c, f*, and *g* sites. In addition, both the N-termini and C-termini of some peptides were selectively modified with EE or KK.

### Preparation of MDV stock

Primary chicken embryo fibroblasts (CEFs) were cultured as previously described [46]. CEF-associated MDV strain RB1B,which was obtained from the Shandong Agriculture University and passaged multiple times in primary CEFs was incubated for 2 hrs at 37°C. Following incubation, the virus inoculum on the cells was replaced with DMEM supplemented with 2% FBS, and the cultures were incubated for another 5 days longer Consistent and uniform plaques were observed and counted using an Olympus microscope, and images were captured using DP Controller. CEF-associated MDV from the same passage at 2×10^4^ plaque forming units (pfu) was used in both infectivity assays in this study.

### Protein expression and purification

*Escherichia coli* strain Ros transformed with the recombinant pGEX-6p-I plasmid was grown at 37°C in 2× YTA to an optical density of 0.8–1.0 (OD at 590 nm) prior to induction with 1 mM IPTG for 4 hrs. Bacterial cells were harvested and lysed by sonication in PBS (pH 7.3). Triton X-100 was then added to a final concentration of 1% and the lysate was incubated for 30 min at 0°C. The clarified supernatants were passed through a Glutathione-Sepharose 4B column. The GST fusion protein-bound column was washed with at least 10 column volumes of PBS and eluted with 3 column volumes of reduced glutathione. The GST fusion proteins were then cleaved by GST fused to rhinovirus 3C protease at 5°C for 16 hrs in 50 mM Tris-HCl, pH 7.0. The cleaved proteins were purified by affinity filtration (with the Glutathione-Sepharose 4B column), and the unbound protein was extracted and concentrated by ultrafiltration through 3 K membranes (Millipore). The resulting proteins were dialyzed against PBS, reduced to the desired concentration by ultrafiltration and stored at −70°C until further analysis. GST fusion proteins and cleaved proteins were analyzed by SDS-PAGE.

### Immunoprecipitation and western blot analysis

MDV-infected or mock-infected cell monolayers were washed 3 times with PBS and resuspended in immunoprecipitation buffer (Applygen Technologies, Inc.) in the presence of a protease inhibitor cocktail (Sigma). The cells were shaken extensively on ice for 5 min and then centrifuged at 12,000×g for 1 min at 4°C. The supernatant was then transferred to fresh tubes. Approximately 1 mg of anti-gH or anti-gB antibodies were added to the cell supernatant with protein A followed by incubation on ice for at least 6 hrs. The same treatment with IgG antibody was performed as a control. The supernatant was removed after centrifugation at 2000××g for 3 min, and the protein A was washed 5 times with PBS. Protein A beads and proteins were analyzed on a 12% SDS-PAGE. The proteins were transferred to a nitrocellulose membrane, which was probed with anti-gB or anti-gH antibodies and subsequently with HRP-conjugated goat anti-rabbit IgG. The experiment was performed in triplicate.

### GST pull-down

The expression plasmid pGEX-6p-1 containing the GST fusion gene was constructed and transfected into *E. coli*. The peptide was incubated with purified GST-fused HR protein or GST protein in the presence of Glutathione-Sepharose 4B beads at 4°C for at least 8 hrs. The beads were washed with PBS 5 times, and the proteins were eluted in 20 mM reduced glutathione. The proteins were analyzed on a 15% SDS-PAGE. The experiment was performed in triplicate.

### Gel filtration analysis

The purified cleaved peptides were loaded onto a Superdex G75 column in 20 mM Tris-HCl, pH 8.0. The peak molecular weight was estimated by comparison with protein standards running on the same column. The peak fractions were collected and analyzed on an 18% SDS-PAGE. The analytical column was calibrated using a series of individual runs of standard molecular weight proteins as markers, including bovine serum albumin (68 kDa), egg white albumin (43 kDa), ribose nucleotidase (13.7 kDa), aprotinin (6.5 kDa), antimicrobial peptides (5 kDa), and vitamin B12 (1.4 kDa).

### Circular dichroism (CD) spectroscopy analyses

The purified peptides were dissolved in 10 μM PBS, pH 7.4 with 25% 2,2,2 trifluoroethanol (TFE) and studied using a CD spectropolarimeter (J-710 Model, JASCO) with 0.2 cm path length cuvettes from 195–245 nm. CD spectrum analysis was performed to study the secondary structural changes in the individual peptides and any combination of two or three peptides (peptide mixture) at an equimolar concentration in PBS with 25% TFE (Sigma-Aldrich, Milan, Italy). For these studies, a single peptide was prepared at a concentration of 30 μM, and peptide mixtures were prepared at equimolar concentrations in a constant volume (for example, for two peptide mixtures, the final concentration of each peptide was 15 μM). The buffers were also filtered in a vacuum pump system using 0.2 μm pore membrane filters. The routine calibration of the machine was performed with D-10-camphorsulfonic acid (60 mg 100 ml^−1^) using the equation [Q]  = 100 Q cnl^−1^ where Q is the ellipticity (mdeg), c is the peptide concentration (mM), n is the number of residues and l is the path length (cm). Data analysis and acquisition were performed using the inbuilt spectra manager software provided with the machine. On average, three scans were performed with a scanning rate of 200 nm min^−1^. The results were expressed as the mean residue ellipticity [Q] (10^−3^ degree cm^2^ dmol^−1^). In addition, thermodynamic stability was measured at 222 nm by recording the CD signal in the temperature range of 20–100°C with a scan rate of 1°C min^−1^.

### Effects of peptides on plaque formation

All peptides were dissolved in DMEM without FBS and used at a range of concentrations. For the antiviral activities of peptides in the co-treatment assay, 100 pfu of MDV was incubated with the peptide at different concentrations for 2 hrs at 37°C. Following incubation, the virus-peptide mixtures on the cells were replaced with DMEM supplemented with 2% FBS, and the cultures were incubated for 5 days. At the end of this period, 50% inhibitory concentrations (IC_50_) values were calculated. To assess the effects of peptides on the inhibition of MDV infectivity, four different methods [27] of treating cell monolayers were used: 1) virus pre-treatment, in which virus was incubated in the presence of peptides at 25 μM for 1 hr at 37 °C and was then titrated on cell monolayers; 2) cell pre-treatment, in which the cells were incubated with peptides for 30 min at 4°C, the peptides were removed, and the cells were washed with PBS and then infected with MDV; 3) co-treatment, in which the cells were incubated with peptides in the presence of viral inoculum for 1 hr at 37°C; and 4) post-treatment, in which the cell monolayers were infected with virus for 45 min at 37°C and the peptides were added to the inoculum followed by an additional 30 min incubation at 37°C. For all treatments, unpenetrated virus was inactivated in a low pH buffer after the 45 min incubation with cells at 37°C. The monolayers were incubated for 5 days at 37°C in DMEM supplemented with 2% FBS. The ratio of plaque counts to the no peptide sample control is reported as the percentage of plaque formation (by arithmetic conversion of the mean percent plaque formation). The results are expressed as the average of triplicates ± the standard deviation, and all experiments were performed in parallel with each peptide and a non-specific peptide (i.e., scramble).

### LDH assay for toxicity analysis

Peptide cytotoxicity was measured by a lactate dehydrogenase (LDH) assay according to the manufacturer's instructions using a cytotoxicity detection kit (Roche).

## Supporting Information

Figure S1 **Predicted the 3D structure of the MDV gB domain.** Predicted the 3D structure of the MDV gB domain by modeling against the known structure of gB using Swiss-Model via the ExPASy web server. Structure of the HSV gB homologs was used to highlight the homologous peptides since gB and gH are relatively well conserved except domain II.
(TIF)

## References

[1] Fukuchi K, Tanaka A, Schierman LW, Witter RL, Nonoyama M (1985) The structure of Marek disease virus DNA: the presence of unique expansion in nonpathogenic viral DNA. Proc Natl Acad Sci U S A 82: 751–754. 2983314 10.1073/pnas.82.3.751 PMC397124

[2] Tulman ER, Afonso CL, Lu Z, Zsak L, Rock DL, et al (2000) The genome of a very virulent Marek's disease virus. J Virol 74: 7980–7988. 10933706 10.1128/jvi.74.17.7980-7988.2000 PMC112329

[3] Burgess SC, Young JR, Baaten BJ, Hunt L, Ross LN, et al (2004) Marek's disease is a natural model for lymphomas overexpressing Hodgkin's disease antigen (CD30). Proc Natl Acad Sci U S A 101: 13879–13884. 15356338 10.1073/pnas.0305789101 PMC518847

[4] Osterrieder N, Kamil JP, Schumacher D, Tischer BK, Trapp S (2006) Marek's disease virus: from miasma to model. Nat Rev Microbiol 4: 283–294. 16541136 10.1038/nrmicro1382

[5] Gimeno IM, Witter RL, Hunt HD, Lee LF, Reddy SM, et al (2001) Marek's disease virus infection in the brain: virus replication, cellular infiltration, and major histocompatibility complex antigen expression. Vet Pathol 38: 491–503. 11572556 10.1354/vp.38-5-491

[6] Li Y, Kang M, Su S, Ding J, Cui Z, et al (2010) Evaluation the immuno-protective effect of an infectious clone of meq- deleted Marek's disease virus. Wei Sheng Wu Xue Bao 50: 942–948. 20815243

[7] Silva RF, Dunn JR, Cheng HH, Niikura M (2010) A MEQ-deleted Marek's disease virus cloned as a bacterial artificial chromosome is a highly efficacious vaccine. Avian Dis 54: 862–869. 20608531 10.1637/9048-090409-Reg.1

[8] Ajithdoss DK, Reddy SM, Suchodolski PF, Lee LF, Kung HJ, et al (2009) In vitro characterization of the Meq proteins of Marek's disease virus vaccine strain CVI988. Virus Res 142: 57–67. 19189855 10.1016/j.virusres.2009.01.008

[9] Harrison SC (2008) Viral membrane fusion. Nat Struct Mol Biol 15: 690–698. 18596815 10.1038/nsmb.1456 PMC2517140

[10] Falanga A, Tarallo R, Vitiello G, Vitiello M, Perillo E, et al (2012) Biophysical characterization and membrane interaction of the two fusion loops of glycoprotein B from herpes simplex type I virus. PLoS One 7: e32186. 22384173 10.1371/journal.pone.0032186 PMC3285657

[11] Connolly SA, Jackson JO, Jardetzky TS, Longnecker R (2011) Fusing structure and function: a structural view of the herpesvirus entry machinery. Nat Rev Microbiol 9: 369–381. 21478902 10.1038/nrmicro2548 PMC3242325

[12] Heldwein EE, Krummenacher C (2008) Entry of herpesviruses into mammalian cells. Cell Mol Life Sci 65: 1653–1668. 18351291 10.1007/s00018-008-7570-z PMC11131758

[13] Muggeridge MI (2000) Characterization of cell-cell fusion mediated by herpes simplex virus 2 glycoproteins gB, gD, gH and gL in transfected cells. J Gen Virol 81: 2017–2027. 10900041 10.1099/0022-1317-81-8-2017

[14] Maurer UE, Sodeik B, Grunewald K (2008) Native 3D intermediates of membrane fusion in herpes simplex virus 1 entry. Proc Natl Acad Sci U S A 105: 10559–10564. 18653756 10.1073/pnas.0801674105 PMC2492464

[15] Atanasiu D, Saw WT, Cohen GH, Eisenberg RJ (2010) Cascade of events governing cell-cell fusion induced by herpes simplex virus glycoproteins gD, gH/gL, and gB. J Virol 84: 12292–12299. 20861251 10.1128/JVI.01700-10 PMC2976417

[16] Farnsworth A, Wisner TW, Webb M, Roller R, Cohen G, et al (2007) Herpes simplex virus glycoproteins gB and gH function in fusion between the virion envelope and the outer nuclear membrane. Proc Natl Acad Sci U S A 104: 10187–10192. 17548810 10.1073/pnas.0703790104 PMC1891206

[17] Maresova L, Pasieka TJ, Grose C (2001) Varicella-zoster Virus gB and gE coexpression, but not gB or gE alone, leads to abundant fusion and syncytium formation equivalent to those from gH and gL coexpression. J Virol 75: 9483–9492. 11533210 10.1128/JVI.75.19.9483-9492.2001 PMC114515

[18] Atanasiu D, Whitbeck JC, Cairns TM, Reilly B, Cohen GH, et al (2007) Bimolecular complementation reveals that glycoproteins gB and gH/gL of herpes simplex virus interact with each other during cell fusion. Proc Natl Acad Sci U S A 104: 18718–18723. 18003913 10.1073/pnas.0707452104 PMC2141843

[19] Avitabile E, Forghieri C, Campadelli-Fiume G (2007) Complexes between herpes simplex virus glycoproteins gD, gB, and gH detected in cells by complementation of split enhanced green fluorescent protein. J Virol 81: 11532–11537. 17670828 10.1128/JVI.01343-07 PMC2045520

[20] Heldwein EE, Lou H, Bender FC, Cohen GH, Eisenberg RJ, et al (2006) Crystal structure of glycoprotein B from herpes simplex virus 1. Science 313: 217–220. 16840698 10.1126/science.1126548

[21] Yoshida S, Lee LF, Yanagida N, Nazerian K (1994) Mutational analysis of the proteolytic cleavage site of glycoprotein B (gB) of Marek's disease virus. Gene 150: 303–306. 7821796 10.1016/0378-1119(94)90442-1

[22] Chowdary TK, Cairns TM, Atanasiu D, Cohen GH, Eisenberg RJ, et al (2010) Crystal structure of the conserved herpesvirus fusion regulator complex gH-gL. Nat Struct Mol Biol 17: 882–888. 20601960 10.1038/nsmb.1837 PMC2921994

[23] Browne HM (2009) The role of glycoprotein H in herpesvirus membrane fusion. Protein Pept Lett 16: 760–765. 19601905 10.2174/092986609788681850

[24] Pertel PE (2002) Human herpesvirus 8 glycoprotein B (gB), gH, and gL can mediate cell fusion. J Vriol 76: 4390–4400. 10.1128/JVI.76.9.4390-4400.2002 PMC155071 11932406

[25] Galdiero S, Vitiello M, D'Isanto M, Falanga A, Collins C, et al (2006) Analysis of synthetic peptides from heptad-repeat domains of herpes simplex virus type 1 glycoproteins H and B. J Gen Virol. 87: 1085–1097. 10.1099/vir.0.81794-0 16603508

[26] Galdiero S, Falanga A, Vitiello M, Browne H, Pedone C, et al (2005) Fusogenic domains in herpes simplex virus type 1 glycoprotein H. J Biol Chem. 280: 28632–28643. 10.1074/jbc.M505196200 15937337

[27] Galdiero S, Vitiello M, D'Isanto M, Falanga A, Cantisani M, et al (2008) The identification and characterization of fusogenic domains in herpes virus glycoprotein B molecules. Chembiochem 9: 758–767. 18311743 10.1002/cbic.200700457

[28] Galdiero S, Falanga A, Vitiello M, D'Isanto M, Cantisani M, et al (2008) Peptides containing membrane-interacting motifs inhibit herpes simplex virus type 1 infectivity. Peptides 29: 1461–1471. 18572274 10.1016/j.peptides.2008.04.022 PMC7172891

[29] Wang X, Chi X, Wang M (2011) Structural characteristics and antiviral activity of multiple peptides derived from MDV glycoproteins B and H. Virol J. 8: 190. 10.1186/1743-422X-8-190 PMC3113977 21518442

[30] Wang XJ, Li CG, Chi XJ, Wang M (2011) Characterisation and evaluation of antiviral recombinant peptides based on the heptad repeat regions of NDV and IBV fusion glycoproteins. Virology 416: 65–74. 21601229 10.1016/j.virol.2011.05.001

[31] Backovic M, Jardetzky TS (2009) Class III viral membrane fusion proteins. Curr Opin Struct Biol 19: 189–196. 19356922 10.1016/j.sbi.2009.02.012 PMC3076093

[32] Colman PM, Lawrence MC (2003) The structural biology of type I viral membrane fusion. Nat Rev Mol Cell Biol 4: 309–319. 12671653 10.1038/nrm1076

[33] Lopper M, Compton T (2004) Coiled-coil domains in glycoproteins B and H are involved in human cytomegalovirus membrane fusion. J Virol 78: 8333–8341. 15254205 10.1128/JVI.78.15.8333-8341.2004 PMC446119

[34] Wu P, Reed WM, Yoshida S, Sui D, Lee LF (1999) Identification and characterization of glycoprotein H of MDV-1 GA strain. Acta Virol 43: 152–158. 10696437

[35] Lee SI, Ohashi K, Sugimoto C, Onuma M (2001) Heparin inhibits plaque formation by cell-free Marek's disease viruses in vitro. J Vet Med Sci 63: 427–432. 11346178 10.1292/jvms.63.427

[36] Eggink D, Berkhout B, Sanders RW (2010) Inhibition of HIV-1 by fusion inhibitors. Curr Pharm Des 16: 3716–3728. 21128887 10.2174/138161210794079218

[37] Liu S, Lu H, Niu J, Xu Y, Wu S, et al (2005) Different from the HIV fusion inhibitor C34, the anti-HIV drug Fuzeon (T-20) inhibits HIV-1 entry by targeting multiple sites in gp41 and gp120. J Biol Chem 280: 11259–11273. 15640162 10.1074/jbc.M411141200

[38] Falanga A, Cantisani M, Pedone C, Galdiero S (2009) Membrane fusion and fission: enveloped viruses. Protein Pept Lett 16: 751–759. 19601904 10.2174/092986609788681760

[39] Chernomordik LV, Kozlov MM (2003) Protein-lipid interplay in fusion and fission of biological membranes. Annu Rev Biochem 72: 175–207. 14527322 10.1146/annurev.biochem.72.121801.161504

[40] Subramanian RP, Geraghty RJ (2007) Herpes simplex virus type 1 mediates fusion through a hemifusion intermediate by sequential activity of glycoproteins D, H, L, and B. Proc Natl Acad Sci U S A. 104: 2903–2908. 10.1073/pnas.0608374104 PMC1815279 17299053

[41] Galdiero S, Falanga A, Vitiello M, D'Isanto M, Collins C, et al (2007) Evidence for a role of the membrane-proximal region of herpes simplex virus Type 1 glycoprotein H in membrane fusion and virus inhibition. Chembiochem 8: 885–895. 17458915 10.1002/cbic.200700044

[42] Galdiero S, Falanga A, Vitiello M, Raiola L, Fattorusso R, et al (2008) Analysis of a membrane interacting region of herpes simplex virus type 1 glycoprotein H. J Biol Chem. 283: 29993–30009. 10.1074/jbc.M803092200 PMC2662066 18678872

[43] Gianni T, Piccoli A, Bertucci C, Campadelli-Fiume G (2006) Heptad repeat 2 in herpes simplex virus 1 gH interacts with heptad repeat 1 and is critical for virus entry and fusion. J Virol 80: 2216–2224. 16474129 10.1128/JVI.80.5.2216-2224.2006 PMC1395405

[44] Gianni T, Fato R, Bergamini C, Lenaz G, Campadelli-Fiume G (2006) Hydrophobic alpha-helices 1 and 2 of herpes simplex virus gH interact with lipids, and their mimetic peptides enhance virus infection and fusion. J Virol 80: 8190–8198. 16873275 10.1128/JVI.00504-06 PMC1563806

[45] Kato K, Jang HK, Izumiya Y, Cai JS, Tsushima Y, et al (1999) Identification of the Marek's disease virus serotype 2 genes homologous to the glycoprotein B (UL27), ICP18.5 (UL28) and major DNA-binding protein (UL29) genes of herpes simplex virus type 1. J Vet Med Sci 61: 1161–1165. 10563297 10.1292/jvms.61.1161

[46] Bissell MJ, Farson D, Tung AS (1977) Cell shape and hexose transport in normal and virus-transformed cells in culture. J Supramol Struct 6: 1–12. 197315 10.1002/jss.400060102

