# Supplementary figures and images for: Interaction Domain of Glycoproteins gB and gH of Marek's Disease Virus and Identification of an Antiviral Peptide with Dual Functions
Source: PLoS One. 2013 Feb 6;8(2):e54761. doi: 10.1371/journal.pone.0054761 (PMC3566115; doi:10.1371/journal.pone.0054761)

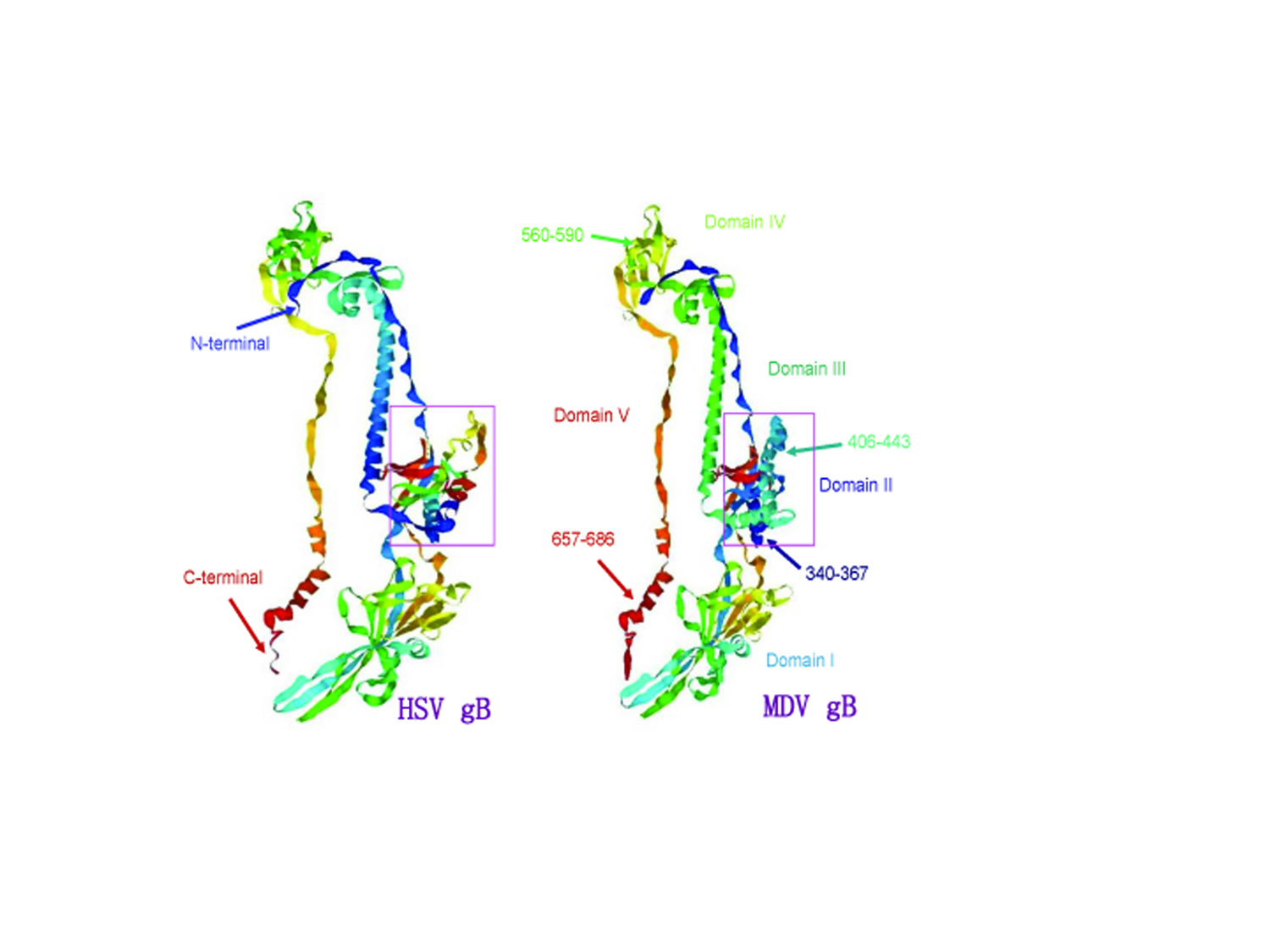

Supplement: Figure S1 — Predicted the 3D structure of the MDV gB domain. Predicted the 3D structure of the MDV gB domain by modeling against the known structure of gB using Swiss-Model via the ExPASy web server. Structure of the HSV gB homologs was used to highlight the homologous peptides since gB and gH are relatively well conserved except domain II. (TIF) [file pone.0054761.s001.tif]
